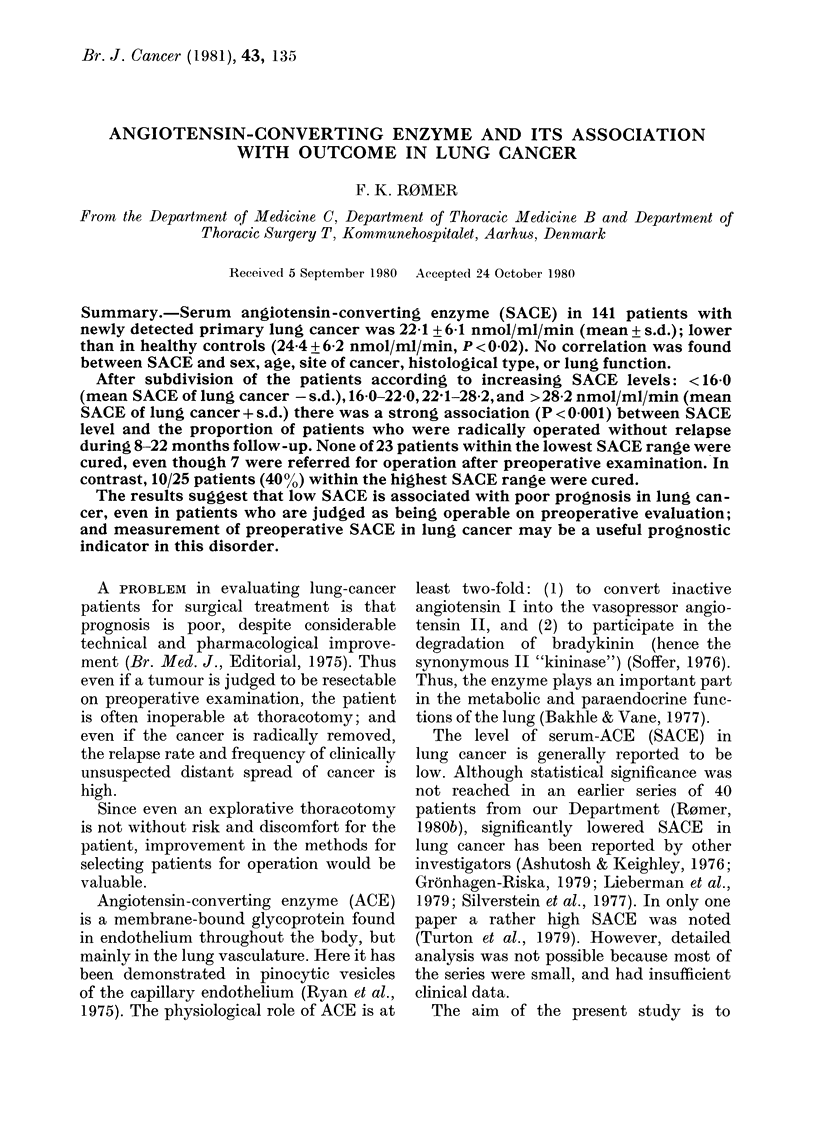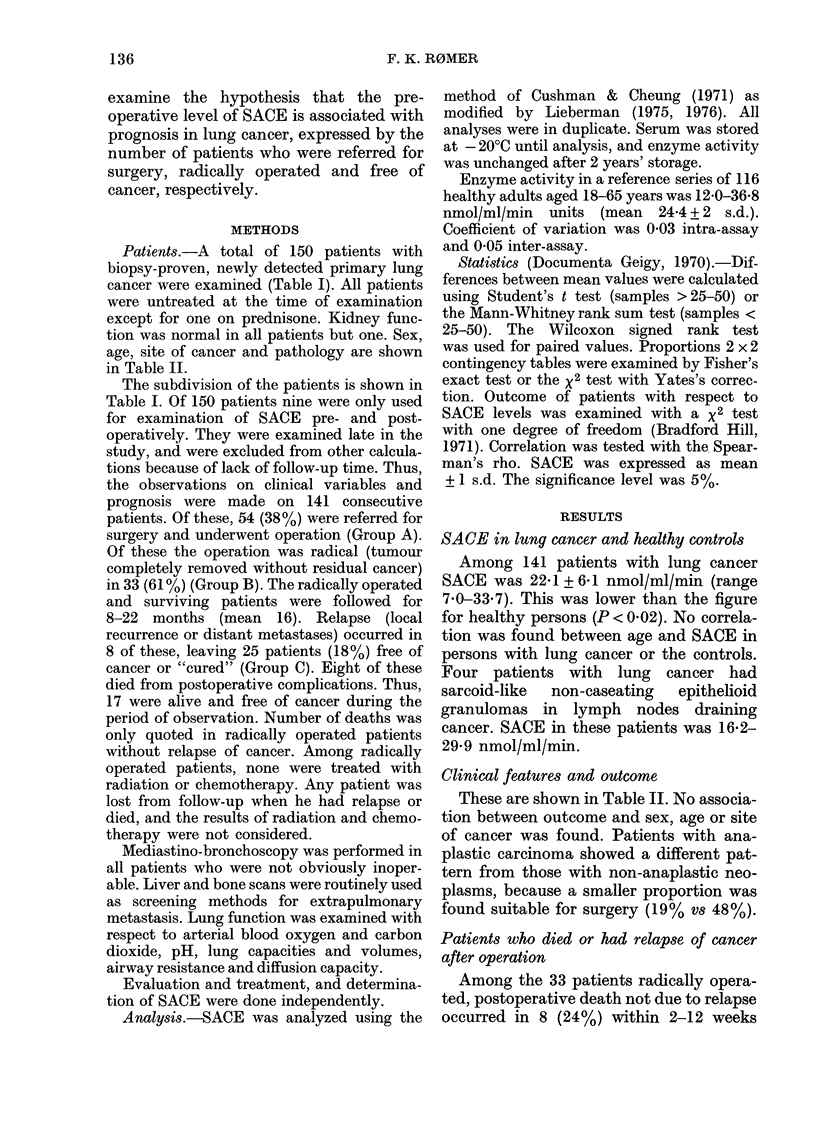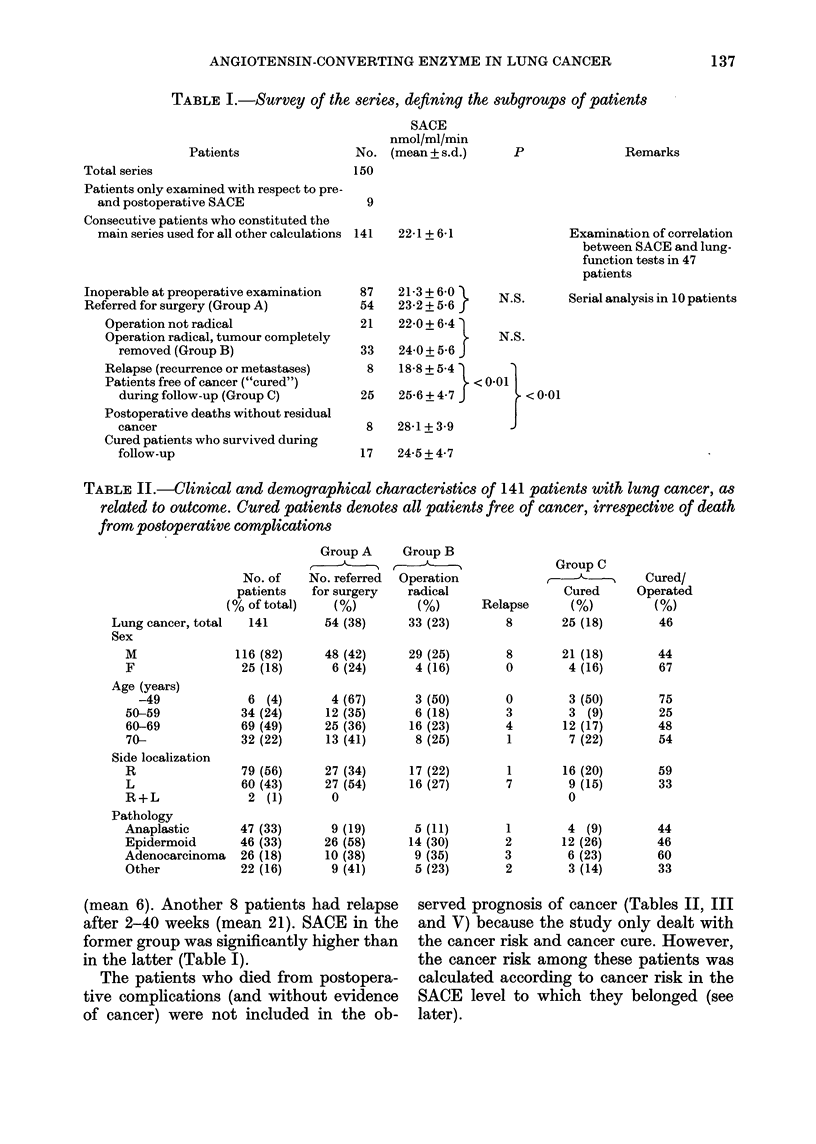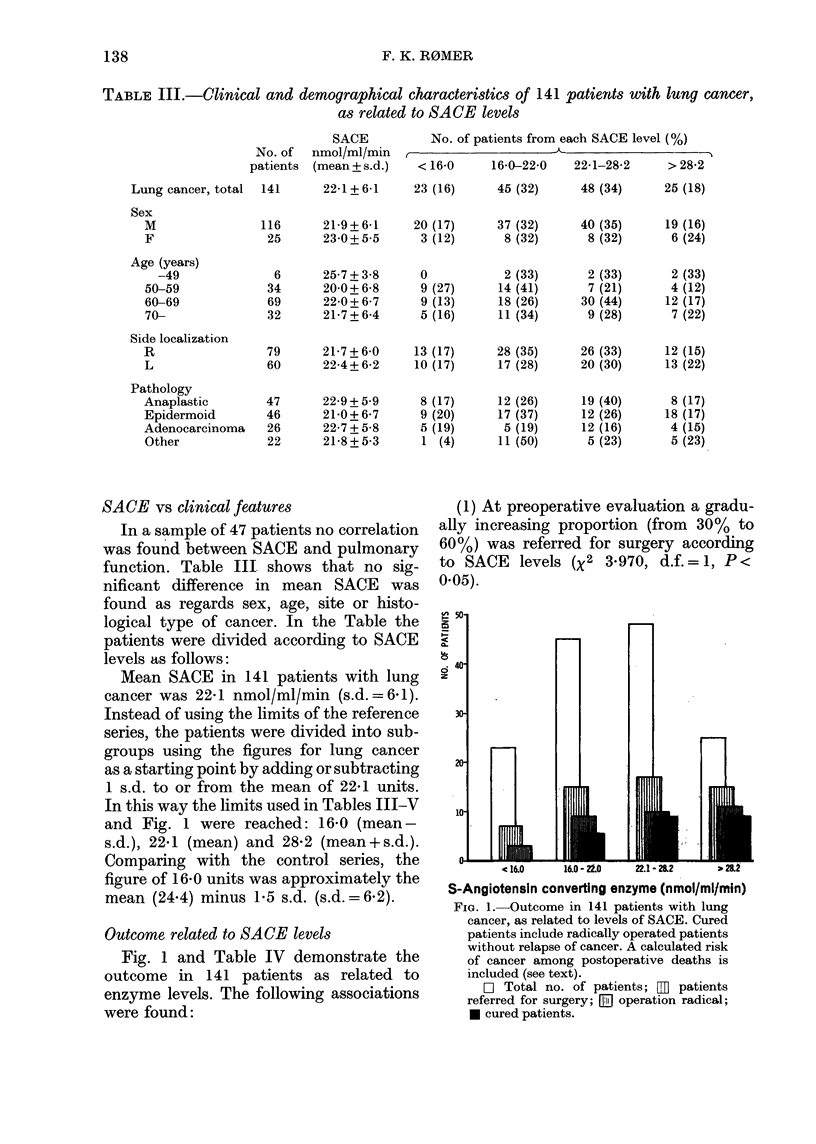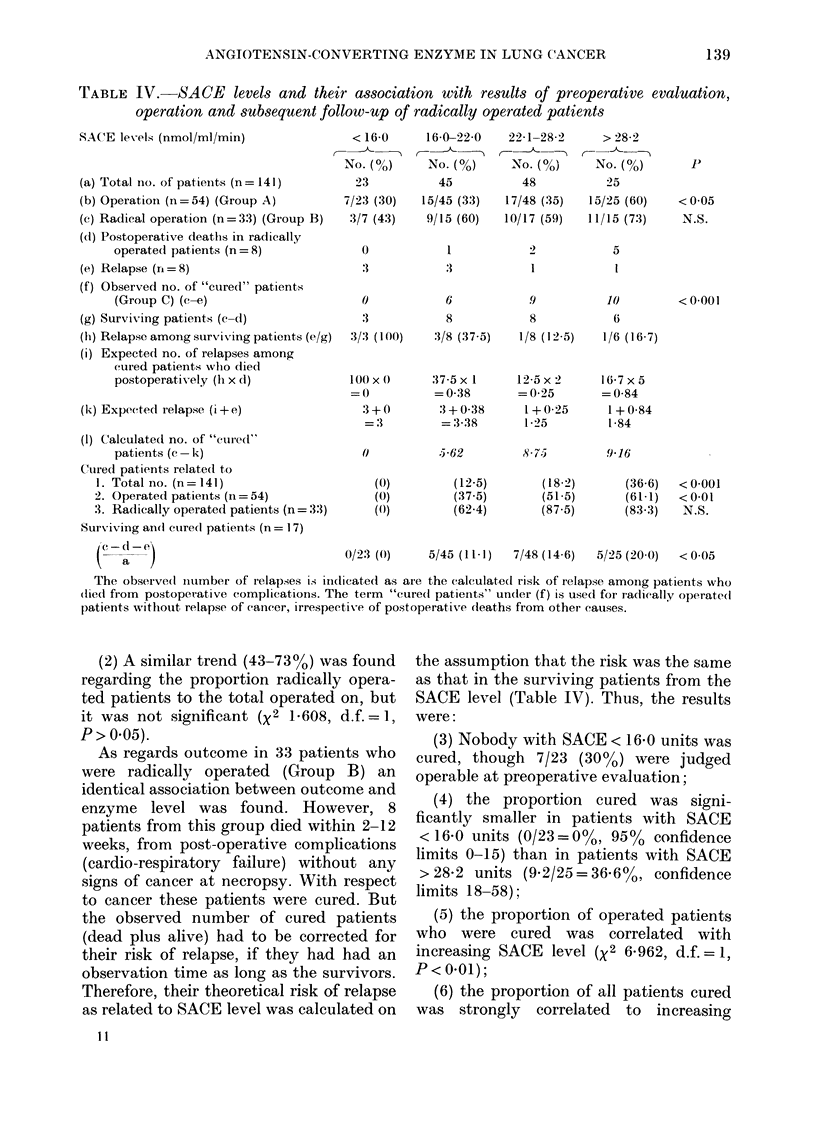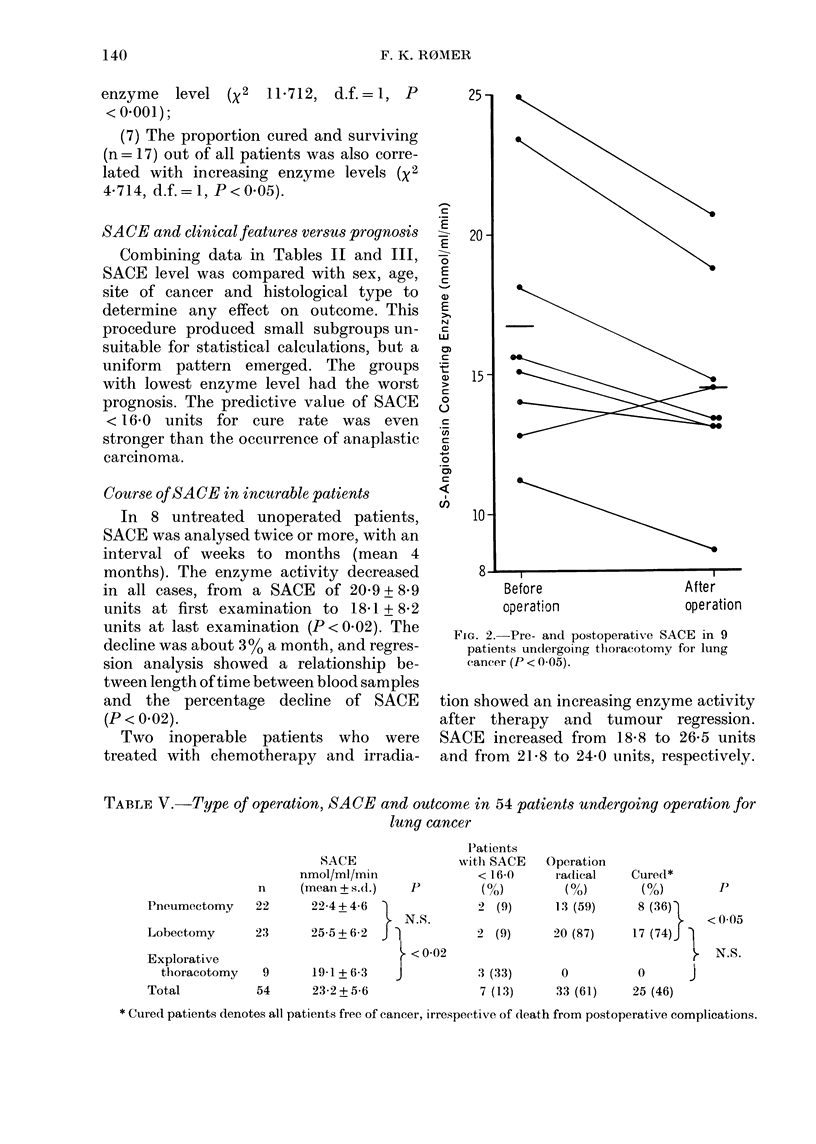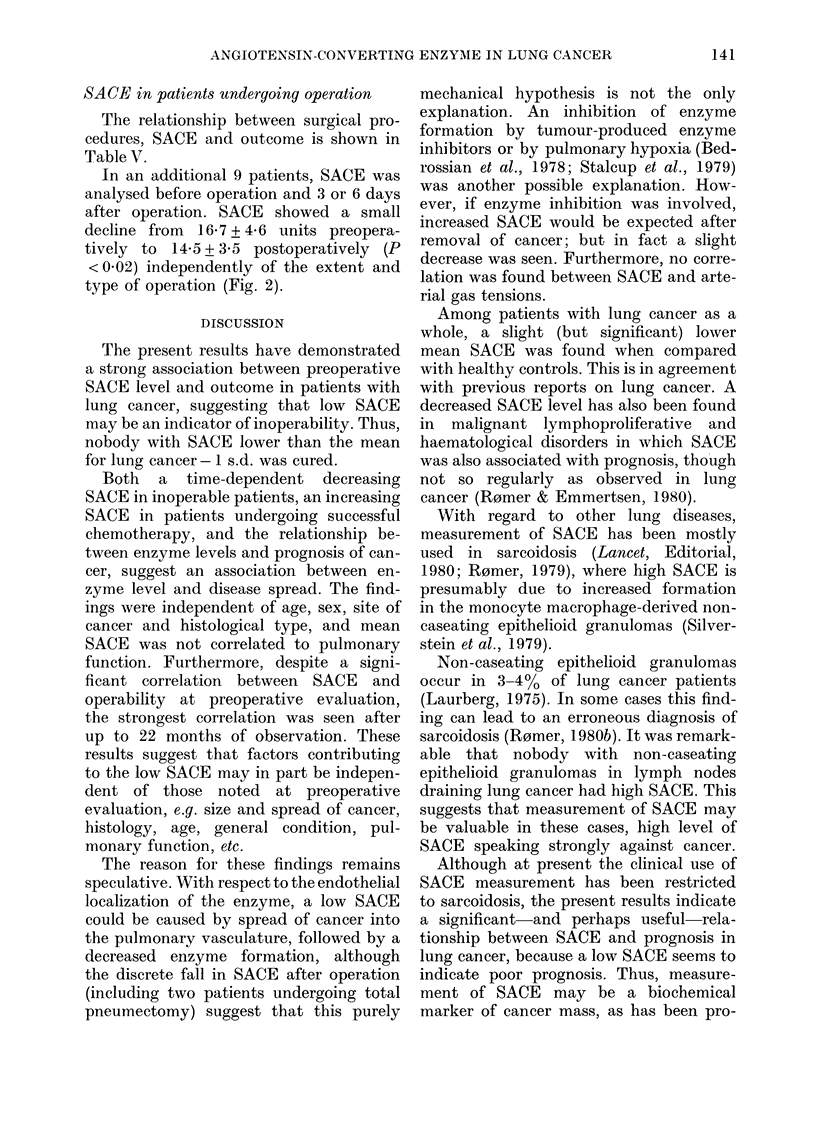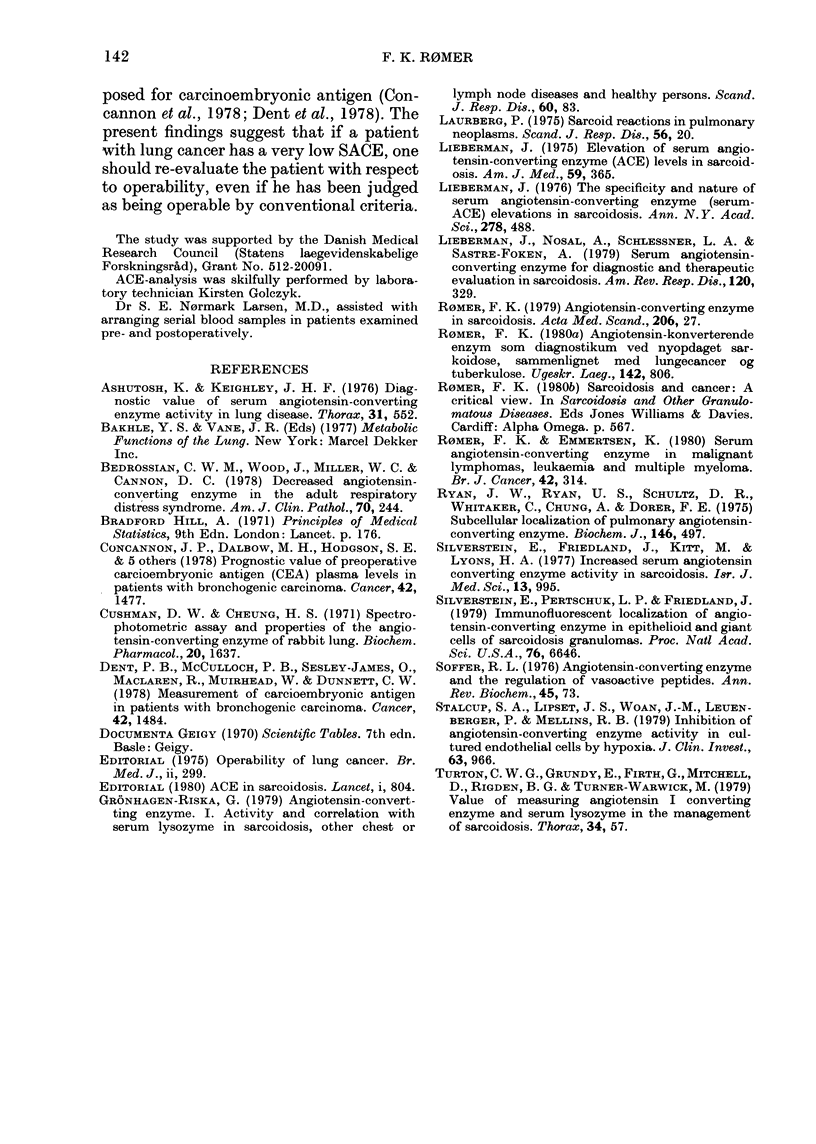# Angiotensin-converting enzyme and its association with outcome in lung cancer.

**DOI:** 10.1038/bjc.1981.21

**Published:** 1981-02

**Authors:** F. K. Rømer

## Abstract

Serum angiotensin-converting enzyme (SACE) in 141 patients with newly detected primary lung cincer was 22.1 +/- 6.1 nmol/ml/min (mean +/- s.d.); lower than in healthy controls (24.4 +/- 6.2 nmol/ml/min, P less than 0.02). No correlation was found between SACE and sex, age, site of cancer, histological type, or lung function. After subdivision of the patients according to increasing SACE levels: less than 16.0 (mean SACE of lung cancer--s.d.), 16.0-22.0, 22.1-28.2 and greater than 28.2 nmol/ml/min (mean SACE of lung cancer + s.d.) there was a strong association (P less than 0.001) between SACE level and the proportion of patients who were radically operated without relapse during 8-22 months follow-up. None of 23 patients within the lowest SACE range were cured, even though 7 were referred for operation after preoperative examination. In contrast, 10/25 patients (40%) within the highest SACE range were cured. The results suggest that low SACE is associated with poor prognosis in lung cancer, even in patients who are judged as being operable on preoperative evaluation; and measurement of preoperative SACE in lug cancer may be a useful prognostic indicator in this disorder.


*Br. J. Cancer* (1981), **43**, 135

### ANGIOTENSIN-CONVERTING ENZYME AND ITS ASSOCIATION WITH OUTCOME IN LUNG CANCER

F. K. RØMER

*From the Department of Medicine C, Department of Thoracic Medicine B and Department of Thoracic Surgery T, Kommunehospitalet, Aarhus, Denmark*

Received 5 September 1980 Accepted 24 October 1980

**Summary.—Serum angiotensin-converting enzyme (SACE) in 141 patients with newly detected primary lung cancer was 22·1 ± 6·1 nmol/ml/min (mean ± s.d.); lower than in healthy controls (24·4 ± 6·2 nmol/ml/min, $P < 0·02$). No correlation was found between SACE and sex, age, site of cancer, histological type, or lung function.**

**After subdivision of the patients according to increasing SACE levels: < 16·0 (mean SACE of lung cancer − s.d.), 16·0–22·0, 22·1–28·2, and > 28·2 nmol/ml/min (mean SACE of lung cancer + s.d.) there was a strong association ($P < 0·001$) between SACE level and the proportion of patients who were radically operated without relapse during 8–22 months follow-up. None of 23 patients within the lowest SACE range were cured, even though 7 were referred for operation after preoperative examination. In contrast, 10/25 patients (40%) within the highest SACE range were cured.**

**The results suggest that low SACE is associated with poor prognosis in lung cancer, even in patients who are judged as being operable on preoperative evaluation; and measurement of preoperative SACE in lung cancer may be a useful prognostic indicator in this disorder.**

A PROBLEM in evaluating lung-cancer patients for surgical treatment is that prognosis is poor, despite considerable technical and pharmacological improvement (*Br. Med. J.*, Editorial, 1975). Thus even if a tumour is judged to be resectable on preoperative examination, the patient is often inoperable at thoracotomy; and even if the cancer is radically removed, the relapse rate and frequency of clinically unsuspected distant spread of cancer is high.

Since even an explorative thoracotomy is not without risk and discomfort for the patient, improvement in the methods for selecting patients for operation would be valuable.

Angiotensin-converting enzyme (ACE) is a membrane-bound glycoprotein found in endothelium throughout the body, but mainly in the lung vasculature. Here it has been demonstrated in pinocytic vesicles of the capillary endothelium (Ryan *et al.*, 1975). The physiological role of ACE is at least two-fold: (1) to convert inactive angiotensin I into the vasopressor angiotensin II, and (2) to participate in the degradation of bradykinin (hence the synonymous II "kininase") (Soffer, 1976). Thus, the enzyme plays an important part in the metabolic and paraendocrine functions of the lung (Bakhle & Vane, 1977).

The level of serum-ACE (SACE) in lung cancer is generally reported to be low. Although statistical significance was not reached in an earlier series of 40 patients from our Department (Rømer, 1980*b*), significantly lowered SACE in lung cancer has been reported by other investigators (Ashutosh & Keighley, 1976; Grönhagen-Riska, 1979; Lieberman *et al.*, 1979; Silverstein *et al.*, 1977). In only one paper a rather high SACE was noted (Turton *et al.*, 1979). However, detailed analysis was not possible because most of the series were small, and had insufficient clinical data.

The aim of the present study is to examine the hypothesis that the preoperative level of SACE is associated with prognosis in lung cancer, expressed by the number of patients who were referred for surgery, radically operated and free of cancer, respectively.

### METHODS

*Patients.*—A total of 150 patients with biopsy-proven, newly detected primary lung cancer were examined (Table I). All patients were untreated at the time of examination except for one on prednisone. Kidney function was normal in all patients but one. Sex, age, site of cancer and pathology are shown in Table II.

The subdivision of the patients is shown in Table I. Of 150 patients nine were only used for examination of SACE pre- and postoperatively. They were examined late in the study, and were excluded from other calculations because of lack of follow-up time. Thus, the observations on clinical variables and prognosis were made on 141 consecutive patients. Of these, 54 (38%) were referred for surgery and underwent operation (Group A). Of these the operation was radical (tumour completely removed without residual cancer) in 33 (61%) (Group B). The radically operated and surviving patients were followed for 8–22 months (mean 16). Relapse (local recurrence or distant metastases) occurred in 8 of these, leaving 25 patients (18%) free of cancer or "cured" (Group C). Eight of these died from postoperative complications. Thus, 17 were alive and free of cancer during the period of observation. Number of deaths was only quoted in radically operated patients without relapse of cancer. Among radically operated patients, none were treated with radiation or chemotherapy. Any patient was lost from follow-up when he had relapse or died, and the results of radiation and chemotherapy were not considered.

Mediastino-bronchoscopy was performed in all patients who were not obviously inoperable. Liver and bone scans were routinely used as screening methods for extrapulmonary metastasis. Lung function was examined with respect to arterial blood oxygen and carbon dioxide, pH, lung capacities and volumes, airway resistance and diffusion capacity.

Evaluation and treatment, and determination of SACE were done independently.

*Analysis.*—SACE was analyzed using the method of Cushman & Cheung (1971) as modified by Lieberman (1975, 1976). All analyses were in duplicate. Serum was stored at $-20°C$ until analysis, and enzyme activity was unchanged after 2 years' storage.

Enzyme activity in a reference series of 116 healthy adults aged 18–65 years was 12·0–36·8 nmol/ml/min units (mean $24·4 \pm 2$ s.d.). Coefficient of variation was 0·03 intra-assay and 0·05 inter-assay.

*Statistics* (Documenta Geigy, 1970).—Differences between mean values were calculated using Student's $t$ test (samples $>25$–50) or the Mann-Whitney rank sum test (samples $<$ 25–50). The Wilcoxon signed rank test was used for paired values. Proportions $2 \times 2$ contingency tables were examined by Fisher's exact test or the $\chi^2$ test with Yates's correction. Outcome of patients with respect to SACE levels was examined with a $\chi^2$ test with one degree of freedom (Bradford Hill, 1971). Correlation was tested with the Spearman's rho. SACE was expressed as mean $\pm 1$ s.d. The significance level was 5%.

### RESULTS

### *SACE in lung cancer and healthy controls*

Among 141 patients with lung cancer SACE was $22·1 \pm 6·1$ nmol/ml/min (range 7·0–33·7). This was lower than the figure for healthy persons ($P < 0·02$). No correlation was found between age and SACE in persons with lung cancer or the controls. Four patients with lung cancer had sarcoid-like non-caseating epithelioid granulomas in lymph nodes draining cancer. SACE in these patients was 16·2–29·9 nmol/ml/min.

### *Clinical features and outcome*

These are shown in Table II. No association between outcome and sex, age or site of cancer was found. Patients with anaplastic carcinoma showed a different pattern from those with non-anaplastic neoplasms, because a smaller proportion was found suitable for surgery (19% *vs* 48%).

### *Patients who died or had relapse of cancer after operation*

Among the 33 patients radically operated, postoperative death not due to relapse occurred in 8 (24%) within 2–12 weeks

TABLE I.—*Survey of the series, defining the subgroups of patients*

| Patients | No. | SACE nmol/ml/min (mean ± s.d.) | *P* | Remarks |
|---|---|---|---|---|
| Total series | 150 | | | |
| Patients only examined with respect to pre- and postoperative SACE | 9 | | | |
| Consecutive patients who constituted the main series used for all other calculations | 141 | 22·1 ± 6·1 | | Examination of correlation between SACE and lung-function tests in 47 patients |
| Inoperable at preoperative examination | 87 | 21·3 ± 6·0 | N.S. | Serial analysis in 10 patients |
| Referred for surgery (Group A) | 54 | 23·2 ± 5·6 | | |
| Operation not radical | 21 | 22·0 ± 6·4 | N.S. | |
| Operation radical, tumour completely removed (Group B) | 33 | 24·0 ± 5·6 | | |
| Relapse (recurrence or metastases) | 8 | 18·8 ± 5·4 | < 0·01; < 0·01 | |
| Patients free of cancer ("cured") during follow-up (Group C) | 25 | 25·6 ± 4·7 | | |
| Postoperative deaths without residual cancer | 8 | 28·1 ± 3·9 | | |
| Cured patients who survived during follow-up | 17 | 24·5 ± 4·7 | | |

TABLE II.—*Clinical and demographical characteristics of* 141 *patients with lung cancer, as related to outcome. Cured patients denotes all patients free of cancer, irrespective of death from postoperative complications*

| | No. of patients (% of total) | Group A: No. referred for surgery (%) | Group B: Operation radical (%) | Relapse | Group C: Cured (%) | Cured/ Operated (%) |
|---|---|---|---|---|---|---|
| Lung cancer, total | 141 | 54 (38) | 33 (23) | 8 | 25 (18) | 46 |
| Sex | | | | | | |
| M | 116 (82) | 48 (42) | 29 (25) | 8 | 21 (18) | 44 |
| F | 25 (18) | 6 (24) | 4 (16) | 0 | 4 (16) | 67 |
| Age (years) | | | | | | |
| –49 | 6 (4) | 4 (67) | 3 (50) | 0 | 3 (50) | 75 |
| 50–59 | 34 (24) | 12 (35) | 6 (18) | 3 | 3 (9) | 25 |
| 60–69 | 69 (49) | 25 (36) | 16 (23) | 4 | 12 (17) | 48 |
| 70– | 32 (22) | 13 (41) | 8 (25) | 1 | 7 (22) | 54 |
| Side localization | | | | | | |
| R | 79 (56) | 27 (34) | 17 (22) | 1 | 16 (20) | 59 |
| L | 60 (43) | 27 (54) | 16 (27) | 7 | 9 (15) | 33 |
| R + L | 2 (1) | 0 | | | 0 | |
| Pathology | | | | | | |
| Anaplastic | 47 (33) | 9 (19) | 5 (11) | 1 | 4 (9) | 44 |
| Epidermoid | 46 (33) | 26 (58) | 14 (30) | 2 | 12 (26) | 46 |
| Adenocarcinoma | 26 (18) | 10 (38) | 9 (35) | 3 | 6 (23) | 60 |
| Other | 22 (16) | 9 (41) | 5 (23) | 2 | 3 (14) | 33 |

(mean 6). Another 8 patients had relapse after 2–40 weeks (mean 21). SACE in the former group was significantly higher than in the latter (Table I).

The patients who died from postoperative complications (and without evidence of cancer) were not included in the observed prognosis of cancer (Tables II, III and V) because the study only dealt with the cancer risk and cancer cure. However, the cancer risk among these patients was calculated according to cancer risk in the SACE level to which they belonged (see later).

TABLE III.—*Clinical and demographical characteristics of* 141 *patients with lung cancer, as related to SACE levels*

| | No. of patients | SACE nmol/ml/min (mean ± s.d.) | No. of patients from each SACE level (%) | | | |
|---|---|---|---|---|---|---|
| | | | < 16·0 | 16·0–22·0 | 22·1–28·2 | > 28·2 |
| Lung cancer, total | 141 | 22·1 ± 6·1 | 23 (16) | 45 (32) | 48 (34) | 25 (18) |
| Sex | | | | | | |
| M | 116 | 21·9 ± 6·1 | 20 (17) | 37 (32) | 40 (35) | 19 (16) |
| F | 25 | 23·0 ± 5·5 | 3 (12) | 8 (32) | 8 (32) | 6 (24) |
| Age (years) | | | | | | |
| –49 | 6 | 25·7 ± 3·8 | 0 | 2 (33) | 2 (33) | 2 (33) |
| 50–59 | 34 | 20·0 ± 6·8 | 9 (27) | 14 (41) | 7 (21) | 4 (12) |
| 60–69 | 69 | 22·0 ± 6·7 | 9 (13) | 18 (26) | 30 (44) | 12 (17) |
| 70– | 32 | 21·7 ± 6·4 | 5 (16) | 11 (34) | 9 (28) | 7 (22) |
| Side localization | | | | | | |
| R | 79 | 21·7 ± 6·0 | 13 (17) | 28 (35) | 26 (33) | 12 (15) |
| L | 60 | 22·4 ± 6·2 | 10 (17) | 17 (28) | 20 (30) | 13 (22) |
| Pathology | | | | | | |
| Anaplastic | 47 | 22·9 ± 5·9 | 8 (17) | 12 (26) | 19 (40) | 8 (17) |
| Epidermoid | 46 | 21·0 ± 6·7 | 9 (20) | 17 (37) | 12 (26) | 18 (17) |
| Adenocarcinoma | 26 | 22·7 ± 5·8 | 5 (19) | 5 (19) | 12 (16) | 4 (15) |
| Other | 22 | 21·8 ± 5·3 | 1 (4) | 11 (50) | 5 (23) | 5 (23) |

*SACE* vs *clinical features*

In a sample of 47 patients no correlation was found between SACE and pulmonary function. Table III shows that no significant difference in mean SACE was found as regards sex, age, site or histological type of cancer. In the Table the patients were divided according to SACE levels as follows:

Mean SACE in 141 patients with lung cancer was 22·1 nmol/ml/min (s.d. = 6·1). Instead of using the limits of the reference series, the patients were divided into subgroups using the figures for lung cancer as a starting point by adding or subtracting 1 s.d. to or from the mean of 22·1 units. In this way the limits used in Tables III–V and Fig. 1 were reached: 16·0 (mean − s.d.), 22·1 (mean) and 28·2 (mean + s.d.). Comparing with the control series, the figure of 16·0 units was approximately the mean (24·4) minus 1·5 s.d. (s.d. = 6·2).

*Outcome related to SACE levels*

Fig. 1 and Table IV demonstrate the outcome in 141 patients as related to enzyme levels. The following associations were found:

(1) At preoperative evaluation a gradually increasing proportion (from 30% to 60%) was referred for surgery according to SACE levels ($\chi^2$ 3·970, d.f. = 1, $P <$ 0·05).

FIG. 1.—Outcome in 141 patients with lung cancer, as related to levels of SACE. Cured patients include radically operated patients without relapse of cancer. A calculated risk of cancer among postoperative deaths is included (see text).

□ Total no. of patients; ▥ patients referred for surgery; ▥ operation radical; ■ cured patients.

TABLE IV.—*SACE levels and their association with results of preoperative evaluation, operation and subsequent follow-up of radically operated patients*

| SACE levels (nmol/ml/min) | < 16·0 | 16·0–22·0 | 22·1–28·2 | > 28·2 | |
|---|---|---|---|---|---|
| | No. (%) | No. (%) | No. (%) | No. (%) | *P* |
| (a) Total no. of patients (n = 141) | 23 | 45 | 48 | 25 | |
| (b) Operation (n = 54) (Group A) | 7/23 (30) | 15/45 (33) | 17/48 (35) | 15/25 (60) | < 0·05 |
| (c) Radical operation (n = 33) (Group B) | 3/7 (43) | 9/15 (60) | 10/17 (59) | 11/15 (73) | N.S. |
| (d) Postoperative deaths in radically operated patients (n = 8) | 0 | 1 | 2 | 5 | |
| (e) Relapse (n = 8) | 3 | 3 | 1 | 1 | |
| (f) Observed no. of "cured" patients (Group C) (c–e) | *0* | *6* | *9* | *10* | < 0·001 |
| (g) Surviving patients (c–d) | 3 | 8 | 8 | 6 | |
| (h) Relapse among surviving patients (e/g) | 3/3 (100) | 3/8 (37·5) | 1/8 (12·5) | 1/6 (16·7) | |
| (i) Expected no. of relapses among cured patients who died postoperatively (h x d) | 100 x 0 = 0 | 37·5 x 1 = 0·38 | 12·5 x 2 = 0·25 | 16·7 x 5 = 0·84 | |
| (k) Expected relapse (i + e) | 3 + 0 = 3 | 3 + 0·38 = 3·38 | 1 + 0·25 1·25 | 1 + 0·84 1·84 | |
| (l) Calculated no. of "cured" patients (c − k) | *0* | *5·62* | *8·75* | *9·16* | |
| Cured patients related to | | | | | |
| 1. Total no. (n = 141) | (0) | (12·5) | (18·2) | (36·6) | < 0·001 |
| 2. Operated patients (n = 54) | (0) | (37·5) | (51·5) | (61·1) | < 0·01 |
| 3. Radically operated patients (n = 33) | (0) | (62·4) | (87·5) | (83·3) | N.S. |
| Surviving and cured patients (n = 17) $\left(\frac{c-d-e}{a}\right)$ | 0/23 (0) | 5/45 (11·1) | 7/48 (14·6) | 5/25 (20·0) | < 0·05 |

The observed number of relapses is indicated as are the calculated risk of relapse among patients who died from postoperative complications. The term "cured patients" under (f) is used for radically operated patients without relapse of cancer, irrespective of postoperative deaths from other causes.

(2) A similar trend (43–73%) was found regarding the proportion radically operated patients to the total operated on, but it was not significant ($\chi^2$ 1·608, d.f. = 1, $P > 0{\cdot}05$).

As regards outcome in 33 patients who were radically operated (Group B) an identical association between outcome and enzyme level was found. However, 8 patients from this group died within 2–12 weeks, from post-operative complications (cardio-respiratory failure) without any signs of cancer at necropsy. With respect to cancer these patients were cured. But the observed number of cured patients (dead plus alive) had to be corrected for their risk of relapse, if they had had an observation time as long as the survivors. Therefore, their theoretical risk of relapse as related to SACE level was calculated on the assumption that the risk was the same as that in the surviving patients from the SACE level (Table IV). Thus, the results were:

(3) Nobody with SACE < 16·0 units was cured, though 7/23 (30%) were judged operable at preoperative evaluation;

(4) the proportion cured was significantly smaller in patients with SACE < 16·0 units (0/23 = 0%, 95% confidence limits 0–15) than in patients with SACE > 28·2 units (9·2/25 = 36·6%, confidence limits 18–58);

(5) the proportion of operated patients who were cured was correlated with increasing SACE level ($\chi^2$ 6·962, d.f. = 1, $P < 0{\cdot}01$);

(6) the proportion of all patients cured was strongly correlated to increasing enzyme level ($\chi^2$ 11·712, d.f. = 1, $P < 0·001$);

(7) The proportion cured and surviving (n = 17) out of all patients was also correlated with increasing enzyme levels ($\chi^2$ 4·714, d.f. = 1, $P < 0·05$).

*SACE and clinical features versus prognosis*

Combining data in Tables II and III, SACE level was compared with sex, age, site of cancer and histological type to determine any effect on outcome. This procedure produced small subgroups unsuitable for statistical calculations, but a uniform pattern emerged. The groups with lowest enzyme level had the worst prognosis. The predictive value of SACE < 16·0 units for cure rate was even stronger than the occurrence of anaplastic carcinoma.

*Course of SACE in incurable patients*

In 8 untreated unoperated patients, SACE was analysed twice or more, with an interval of weeks to months (mean 4 months). The enzyme activity decreased in all cases, from a SACE of 20·9 ± 8·9 units at first examination to 18·1 ± 8·2 units at last examination ($P < 0·02$). The decline was about 3% a month, and regression analysis showed a relationship between length of time between blood samples and the percentage decline of SACE ($P < 0·02$).

Two inoperable patients who were treated with chemotherapy and irradiation showed an increasing enzyme activity after therapy and tumour regression. SACE increased from 18·8 to 26·5 units and from 21·8 to 24·0 units, respectively.

FIG. 2.—Pre- and postoperative SACE in 9 patients undergoing thoracotomy for lung cancer ($P < 0·05$).

TABLE V.—*Type of operation, SACE and outcome in 54 patients undergoing operation for lung cancer*

| | n | SACE nmol/ml/min (mean ± s.d.) | P | Patients with SACE < 16·0 (%) | Operation radical (%) | Cured* (%) | P |
|---|---|---|---|---|---|---|---|
| Pneumectomy | 22 | 22·4 ± 4·6 | } N.S. (Pneumectomy vs Lobectomy) | 2 (9) | 13 (59) | 8 (36) | } < 0·05 (Pneumectomy vs Lobectomy) |
| Lobectomy | 23 | 25·5 ± 6·2 | } < 0·02 (Lobectomy vs Explorative thoracotomy) | 2 (9) | 20 (87) | 17 (74) | } N.S. (Lobectomy vs Explorative thoracotomy) |
| Explorative thoracotomy | 9 | 19·1 ± 6·3 | | 3 (33) | 0 | 0 | |
| Total | 54 | 23·2 ± 5·6 | | 7 (13) | 33 (61) | 25 (46) | |

* Cured patients denotes all patients free of cancer, irrespective of death from postoperative complications.

*SACE in patients undergoing operation*

The relationship between surgical procedures, SACE and outcome is shown in Table V.

In an additional 9 patients, SACE was analysed before operation and 3 or 6 days after operation. SACE showed a small decline from $16{\cdot}7 \pm 4{\cdot}6$ units preoperatively to $14{\cdot}5 \pm 3{\cdot}5$ postoperatively ($P < 0{\cdot}02$) independently of the extent and type of operation (Fig. 2).

### DISCUSSION

The present results have demonstrated a strong association between preoperative SACE level and outcome in patients with lung cancer, suggesting that low SACE may be an indicator of inoperability. Thus, nobody with SACE lower than the mean for lung cancer − 1 s.d. was cured.

Both a time-dependent decreasing SACE in inoperable patients, an increasing SACE in patients undergoing successful chemotherapy, and the relationship between enzyme levels and prognosis of cancer, suggest an association between enzyme level and disease spread. The findings were independent of age, sex, site of cancer and histological type, and mean SACE was not correlated to pulmonary function. Furthermore, despite a significant correlation between SACE and operability at preoperative evaluation, the strongest correlation was seen after up to 22 months of observation. These results suggest that factors contributing to the low SACE may in part be independent of those noted at preoperative evaluation, *e.g.* size and spread of cancer, histology, age, general condition, pulmonary function, *etc.*

The reason for these findings remains speculative. With respect to the endothelial localization of the enzyme, a low SACE could be caused by spread of cancer into the pulmonary vasculature, followed by a decreased enzyme formation, although the discrete fall in SACE after operation (including two patients undergoing total pneumectomy) suggest that this purely mechanical hypothesis is not the only explanation. An inhibition of enzyme formation by tumour-produced enzyme inhibitors or by pulmonary hypoxia (Bedrossian *et al.*, 1978; Stalcup *et al.*, 1979) was another possible explanation. However, if enzyme inhibition was involved, increased SACE would be expected after removal of cancer; but in fact a slight decrease was seen. Furthermore, no correlation was found between SACE and arterial gas tensions.

Among patients with lung cancer as a whole, a slight (but significant) lower mean SACE was found when compared with healthy controls. This is in agreement with previous reports on lung cancer. A decreased SACE level has also been found in malignant lymphoproliferative and haematological disorders in which SACE was also associated with prognosis, though not so regularly as observed in lung cancer (Rømer & Emmertsen, 1980).

With regard to other lung diseases, measurement of SACE has been mostly used in sarcoidosis (*Lancet*, Editorial, 1980; Rømer, 1979), where high SACE is presumably due to increased formation in the monocyte macrophage-derived non-caseating epithelioid granulomas (Silverstein *et al.*, 1979).

Non-caseating epithelioid granulomas occur in 3–4% of lung cancer patients (Laurberg, 1975). In some cases this finding can lead to an erroneous diagnosis of sarcoidosis (Rømer, 1980*b*). It was remarkable that nobody with non-caseating epithelioid granulomas in lymph nodes draining lung cancer had high SACE. This suggests that measurement of SACE may be valuable in these cases, high level of SACE speaking strongly against cancer.

Although at present the clinical use of SACE measurement has been restricted to sarcoidosis, the present results indicate a significant—and perhaps useful—relationship between SACE and prognosis in lung cancer, because a low SACE seems to indicate poor prognosis. Thus, measurement of SACE may be a biochemical marker of cancer mass, as has been proposed for carcinoembryonic antigen (Concannon *et al.*, 1978; Dent *et al.*, 1978). The present findings suggest that if a patient with lung cancer has a very low SACE, one should re-evaluate the patient with respect to operability, even if he has been judged as being operable by conventional criteria.

The study was supported by the Danish Medical Research Council (Statens laegevidenskabelige Forskningsråd), Grant No. 512-20091.

ACE-analysis was skilfully performed by laboratory technician Kirsten Golczyk.

Dr S. E. Nørmark Larsen, M.D., assisted with arranging serial blood samples in patients examined pre- and postoperatively.

### REFERENCES

Ashutosh, K. & Keighley, J. H. F. (1976) Diagnostic value of serum angiotensin-converting enzyme activity in lung disease. *Thorax*, **31**, 552.

Bakhle, Y. S. & Vane, J. R. (Eds) (1977) *Metabolic Functions of the Lung*. New York: Marcel Dekker Inc.

Bedrossian, C. W. M., Wood, J., Miller, W. C. & Cannon, D. C. (1978) Decreased angiotensin-converting enzyme in the adult respiratory distress syndrome. *Am. J. Clin. Pathol.*, **70**, 244.

Bradford Hill, A. (1971) *Principles of Medical Statistics*, 9th Edn. London: Lancet. p. 176.

Concannon, J. P., Dalbow, M. H., Hodgson, S. E. & 5 others (1978) Prognostic value of preoperative carcioembryonic antigen (CEA) plasma levels in patients with bronchogenic carcinoma. *Cancer*, **42**, 1477.

Cushman, D. W. & Cheung, H. S. (1971) Spectrophotometric assay and properties of the angiotensin-converting enzyme of rabbit lung. *Biochem. Pharmacol.*, **20**, 1637.

Dent, P. B., McCulloch, P. B., Sesley-James, O., Maclaren, R., Muirhead, W. & Dunnett, C. W. (1978) Measurement of carcioembryonic antigen in patients with bronchogenic carcinoma. *Cancer*, **42**, 1484.

Documenta Geigy (1970) *Scientific Tables*. 7th edn. Basle: Geigy.

Editorial (1975) Operability of lung cancer. *Br. Med. J.*, ii, 299.

Editorial (1980) ACE in sarcoidosis. *Lancet*, i, 804.

Grönhagen-Riska, G. (1979) Angiotensin-convertting enzyme. I. Activity and correlation with serum lysozyme in sarcoidosis, other chest or lymph node diseases and healthy persons. *Scand. J. Resp. Dis.*, **60**, 83.

Laurberg, P. (1975) Sarcoid reactions in pulmonary neoplasms. *Scand. J. Resp. Dis.*, **56**, 20.

Lieberman, J. (1975) Elevation of serum angiotensin-converting enzyme (ACE) levels in sarcoidosis. *Am. J. Med.*, **59**, 365.

Lieberman, J. (1976) The specificity and nature of serum angiotensin-converting enzyme (serum-ACE) elevations in sarcoidosis. *Ann. N.Y. Acad. Sci.*, **278**, 488.

Lieberman, J., Nosal, A., Schlessner, L. A. & Sastre-Foken, A. (1979) Serum angiotensin-converting enzyme for diagnostic and therapeutic evaluation in sarcoidosis. *Am. Rev. Resp. Dis.*, **120**, 329.

Rømer, F. K. (1979) Angiotensin-converting enzyme in sarcoidosis. *Acta Med. Scand.*, **206**, 27.

Rømer, F. K. (1980*a*) Angiotensin-konverterende enzym som diagnostikum ved nyopdaget sarkoidose, sammenlignet med lungecancer og tuberkulose. *Ugeskr. Laeg.*, **142**, 806.

Rømer, F. K. (1980*b*) Sarcoidosis and cancer: A critical view. In *Sarcoidosis and Other Granulomatous Diseases*. Eds Jones Williams & Davies. Cardiff: Alpha Omega. p. 567.

Rømer, F. K. & Emmertsen, K. (1980) Serum angiotensin-converting enzyme in malignant lymphomas, leukaemia and multiple myeloma. *Br. J. Cancer*, **42**, 314.

Ryan, J. W., Ryan, U. S., Schultz, D. R., Whitaker, C., Chung, A. & Dorer, F. E. (1975) Subcellular localization of pulmonary angiotensin-converting enzyme. *Biochem. J.*, **146**, 497.

Silverstein, E., Friedland, J., Kitt, M. & Lyons, H. A. (1977) Increased serum angiotensin converting enzyme activity in sarcoidosis. *Isr. J. Med. Sci.*, **13**, 995.

Silverstein, E., Pertschuk, L. P. & Friedland, J. (1979) Immunofluorescent localization of angiotensin-converting enzyme in epithelioid and giant cells of sarcoidosis granulomas. *Proc. Natl Acad. Sci. U.S.A.*, **76**, 6646.

Soffer, R. L. (1976) Angiotensin-converting enzyme and the regulation of vasoactive peptides. *Ann. Rev. Biochem.*, **45**, 73.

Stalcup, S. A., Lipset, J. S., Woan, J.-M., Leuenberger, P. & Mellins, R. B. (1979) Inhibition of angiotensin-converting enzyme activity in cultured endothelial cells by hypoxia. *J. Clin. Invest.*, **63**, 966.

Turton, C. W. G., Grundy, E., Firth, G., Mitchell, D., Rigden, B. G. & Turner-Warwick, M. (1979) Value of measuring angiotensin I converting enzyme and serum lysozyme in the management of sarcoidosis. *Thorax*, **34**, 57.